# Current and Emerging Technologies for Probing Molecular Signatures of Traumatic Brain Injury

**DOI:** 10.3389/fneur.2017.00450

**Published:** 2017-08-30

**Authors:** Ari Ercole, Sandra Magnoni, Gloria Vegliante, Roberta Pastorelli, Jakub Surmacki, Sarah Elizabeth Bohndiek, Elisa R. Zanier

**Affiliations:** ^1^Division of Anaesthesia, University of Cambridge, Addenbrooke’s Hospital, Cambridge, United Kingdom; ^2^Department of Anesthesiology and Intensive Care, Fondazione IRCCS Cà Granda, Ospedale Maggiore Policlinico, Milan, Italy; ^3^Laboratory of Acute Brain Injury and Therapeutic Strategies, Department of Neuroscience, IRCCS – Istituto di Ricerche Farmacologiche Mario Negri, Milan, Italy; ^4^Unit of Gene and Protein Biomarkers, Laboratory of Mass Spectrometry, IRCCS – Istituto di Ricerche Farmacologiche Mario Negri, Milan, Italy; ^5^Department of Physics, University of Cambridge, Cambridge, United Kingdom; ^6^Cancer Research UK Cambridge Institute, University of Cambridge, Cambridge, United Kingdom

**Keywords:** traumatic brain injury, microdialysis, proteomics, lipidomics, metabolomics, Raman spectroscopy

## Abstract

Traumatic brain injury (TBI) is understood as an interplay between the initial injury, subsequent secondary injuries, and a complex host response all of which are highly heterogeneous. An understanding of the underlying biology suggests a number of windows where mechanistically inspired interventions could be targeted. Unfortunately, biologically plausible therapies have to-date failed to translate into clinical practice. While a number of stereotypical pathways are now understood to be involved, current clinical characterization is too crude for it to be possible to characterize the biological phenotype in a truly mechanistically meaningful way. In this review, we examine current and emerging technologies for fuller biochemical characterization by the simultaneous measurement of multiple, diverse biomarkers. We describe how clinically available techniques such as cerebral microdialysis can be leveraged to give mechanistic insights into TBI pathobiology and how multiplex proteomic and metabolomic techniques can give a more complete description of the underlying biology. We also describe spatially resolved label-free multiplex techniques capable of probing structural differences in chemical signatures. Finally, we touch on the bioinformatics challenges that result from the acquisition of such large amounts of chemical data in the search for a more mechanistically complete description of the TBI phenotype.

## Introduction

In broad terms, we understand the biology of traumatic brain injury (TBI) as being the result of an interplay between the biomechanics of the initial insult and resulting injury, the effects of the subsequent resuscitative treatments and the host response. These latter factors are important; even the best available prognostic models predict only a small part of the observed variability in outcome (1). The host response is at least in part genetically determined and a number of stereotypical molecular mechanisms are implicated in the pathobiology of TBI (2). Nevertheless, the patient population is highly heterogeneous and our understanding of the biochemistry of how traumatic lesions go on to influence neural connectivity and brain function is very incomplete. An important related issue is the translational gap: time and again successful experimental interventions in animal models have failed to translate into clinical therapies. Even accepted resuscitative and intensive care strategies are blunt instruments with a wide range of harmful side effects (2). A more nuanced understanding of pathobiology is crucial if novel therapeutics and individualized treatment strategies are to be realized.

**Figure 1:**
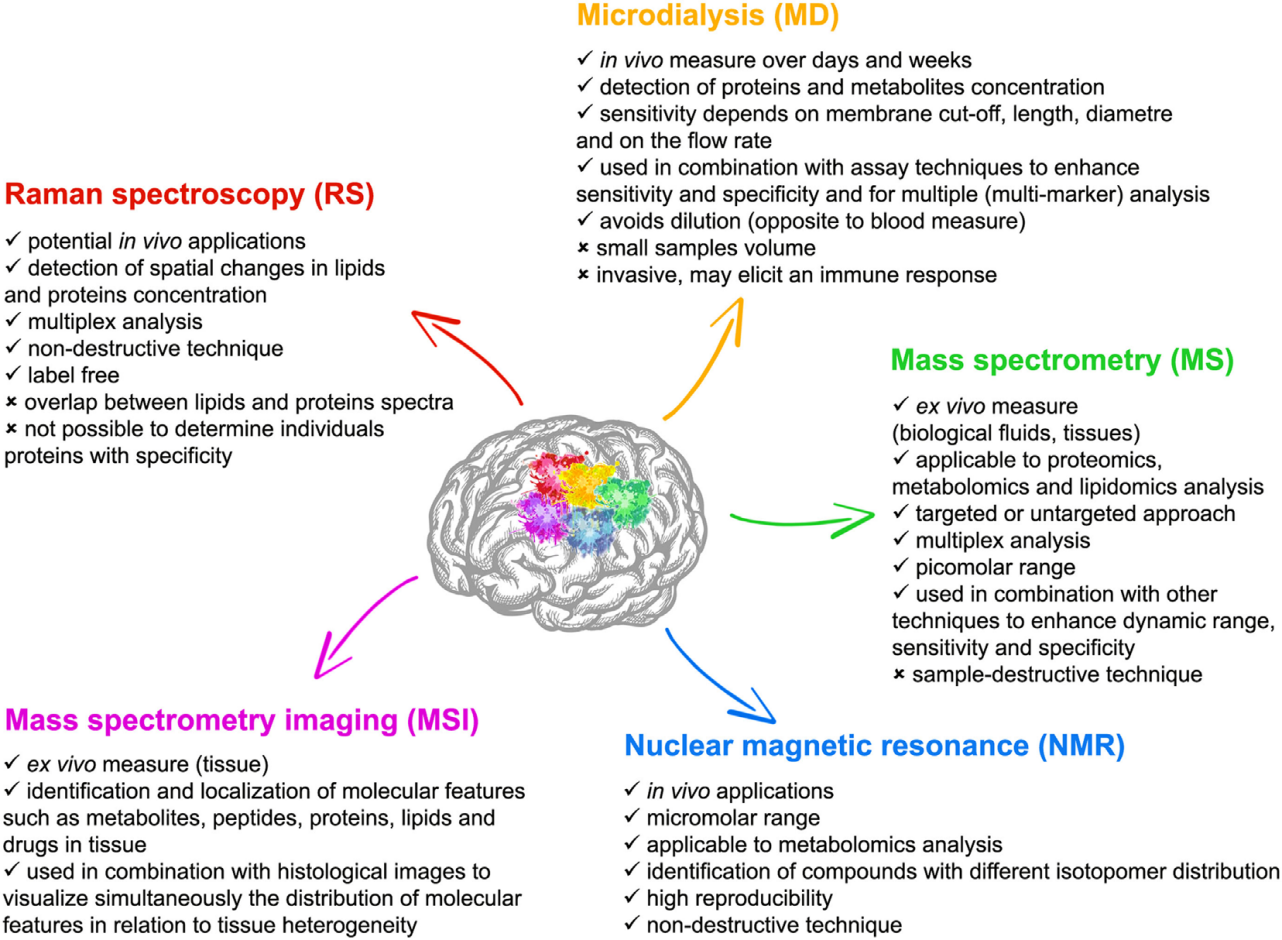
Features of the five main experimental techniques with multiplex chemical sensing capability described in this review; microdialysis (MD), mass spectrometry (MS) [and mass spectrometry imaging (MSI)], nuclear magnetic resonance (NMR), and Raman spectroscopy (RS).

One possible explanation for the translational failures to date is that our understanding of the detailed pathological mechanisms is incomplete or that our clinical phenotype is insufficiently refined to meaningfully characterize the biological state of the patient in a therapeutically meaningful way. Parallel exploration of mechanisms in the clinical setting through direct longitudinal focal monitoring and neuroimaging, and in the laboratory is essential to understand the heterogeneity of TBI, identify biomarkers of injury evolution/restoration, refine experimental models, and develop neurorestorative treatments. A large number of techniques are now available for assessing molecular patterns of TBI in both the experimental and clinical setting (Figure 1). Current technologies, including microdialysis (MD) and various chemically sensitive imaging techniques [such as magnetic resonance (MR) spectroscopy and positron emission tomography] may overlap with emerging technologies such as proteomic and metabolomic approaches or optical spectroscopy.

The molecular signatures generated by emerging experimental techniques are complex and new approaches will likely be needed to make sense of the large amount of data that are becoming available. However, in the post-genomic era, there has been an explosion in technologies exploiting informatics approaches to make the interpretation of biological “big data” tractable. These have had a huge impact on the discovery of both next-generation diagnostics and also new biomarkers for precision medicine approaches to research and treatment. The fundamental premise is that the evolutionary complexity of biological systems renders them difficult to comprehensively understand using only a reductionist approach. Such complexity can become tractable with the use of ’*omics* research; this term implies the study of entities in aggregate. Similar considerations apply to making sense of the large volumes of experimental data provided by novel biochemically sensitive techniques.

We will review the kinds of biochemical data that can now be accessed experimentally and explore possible future trends that will allow a better understanding of mechanisms and timing of disease progression and pave the way for personalized medicine in the treatment of TBI.

## Multiple-Analyte Applications of Cerebral MD

### Technical Aspects

Microdialysis allows continuous sampling of endogenous substances in the interstitial fluid of a target organ (e.g., the brain). The principles of MD have been extensively reviewed elsewhere [for example, Ref. (3)]. Briefly, perfusion fluid is pumped through the inner tube of the MD probe, which has a concentric design and is semipermeable. At the tip of the probe, substances in the interstitial fluid of the target tissue can diffuse along the concentration gradient to some extent equilibrating with the perfusion fluid (4). The resulting dialysate is collected externally in vials.

Compared with the other technologies for assessing molecular patterns of TBI described in this review, MD offers several practical advantages. Most significantly, compounds can be measured semi-continuously *in vivo* over several hours, days, or even for weeks. In addition, MD captures the molecules in the interstitial space at the site of their cellular actions and is, therefore, not subject to dilution such as occurs when sampling other fluids such as blood or cerebrospinal fluid (CSF). MD technology still has its limitations, including its focal resolution since MD samples very small volumes of tissue and its invasiveness [although the effects of catheter microtrauma are transient and reversible and likely of little effect compared to pathological processes (5)]. Moreover, MD may elicit an immune response resulting in altered microcirculation, blood–brain barrier (BBB) damage and perturbation of brain metabolism (3, 6). Another potential limitation of MD is relative substance recovery, an inherent aspect of MD sampling, which implies that substance concentration in the MD samples represent only a fraction of the total amount in the extracellular space. Recovery depends largely on perfusion flow rate (the lower the flow rate, the higher the recovery) and the length and diameter of the membrane. Other factors related to recovery are the diffusion coefficients of the various substances and their interaction with the dialysis membrane (3). At the typically low flow rates used in human MD studies (0.3 µL/min), the sampling time is sufficient for complete equilibration between the perfusion fluid and the surrounding environment to occur: recovery of small molecular weight substances, such as neurotransmitters and energetic compounds (i.e., glucose, lactate, and pyruvate) is very good. However, larger molecules, such as cytokines, chemokines, and other CNS proteins (e.g., axonal proteins), tend to have lower relative recoveries (7, 8). Sensitive analytical methods (i.e., immunoassays) are required to counteract such poor analyte recovery and small sample volumes, another potential limitation of MD. Nonetheless, protein sampling is challenging, and methods to optimize protein recovery with high cut-off membranes are under investigation. Serum albumin, which is widely used in the perfusion fluid, may be used to reduce non-specific binding of the proteins of interests to tubing and membranes, allowing consistent protein recovery (7). Another approach to improve MD recovery is to coat the membranes with substances (i.e., pluronic), again aimed at reducing protein binding (9, 10).

Over time, the MD catheter surface becomes coated with a biofilm due to an inevitable stereotyped inflammatory response to indwelling implants, and this has been demonstrated after 8 days by electron microscopy (11). As a result, a drop-off in recovery *in vivo* may be expected (12). However, such a reduction in performance over time has not been consistently demonstrated (11) and clinical use over prolonged periods is commonplace. The measurement of a small diffusible molecule such as urea whose plasma concentration can be conveniently tracked and is related to brain interstitial concentration has been suggested as an indicator of MD performance (13).

While MD is relatively safe, it is a highly invasive technique and is, therefore, intrinsically unsuitable for use in mild TBI. For similar reasons, it is difficult to obtain normative data from truly “normal” brain that limits our physiological understanding although MD has been applied to patients who have undergone elective neurosurgery in an attempt to obtain better reference ranges (14).

### Clinical and Translational (Research) Applications of MD

Microdialysis was originally developed to measure cerebral neurotransmitters (15). Over the last decade, cerebral MD has been increasingly used in the clinical setting as an established technique for continuous monitoring of substrate delivery and metabolism in patients with TBI and other acute brain injuries. In addition, MD continues to be a powerful translational research tool to explore cerebral physiological and pathophysiological mechanisms. This topic will be the main focus of this section and cited studies are summarized in Table 1.

**Table 1:** Research applications of MD.

Reference	Patients characteristics	Time (after injury)	Biosample	Techniques employed	Key findings
Magnoni et al. (8)	TBI, GCS <8 (16 TBI, no controls)	12–96 h	ECF (contusional and normal appearing brain)	100 kDa MD + serial ELISA (no sample pooling)	Elevated tau in brain ECF correlates with reduced amyloid-β levels and predicts adverse clinical outcome. Tau and NFL levels were 4-fold higher in patients with MD catheters placed in pericontusional regions than those with MD in normal-appearing frontal lobe
Magnoni et al. (5)	TBI, GCS <8, no controls	13–96 h	ECF (contusional and normal appearing brain)	100 kDa MD + serial ELISA (no sample pooling)	Acute tau in brain ECF correlated with DTI measurements of reduced brain white matter integrity in white matter-masked region near the MD catheter
Helmy et al. (7)	TBI, GCS <8 (12 TBI, no controls)	<96 h–5 d	ECF, plasma	100 kDa MD + Multiplex ELISA (sample pooling)	Cytokine production is highly compartmentalized, with quantitative and qualitative differences between brain parenchymal and systemic cytokine concentrations
Helmy et al. (16)	TBI, GCS <8 (20 TBI, no controls)	24 h–5 d	ECF (normal appearing brain), plasma	reverse 100 kDa MD + Multiplex ELISA (sample pooling)	Subcutaneously administered rhIL-1Ra results in a large increase in concentration of this cytokine both in the circulation and in the brain ECF, in TBI patients. rhIL-1Ra treatment modulates the brain extracellular cytokine and chemokine profile
Marklund et al. (17)	TBI, GCS <8 (8 TBI, no controls)	1–5 d	ECF	100 kDa MD + standard ELISA (sample pooling)	Patients with a predominantly focal lesion show higher ECF tau than those with DAI, 1–3 d post injury. Patients with DAI show consistently higher amyloid-β42 levels than those with focal injury
Guilfoyle et al. (18)	TBI, GCS < 8 (12 TBI, controls: paired catheteters in normal appearing brain)	24–48 h to 5 d	ECF (contusional and normal appearing brain)	100 kDa MD + Multiplex ELISA (sample pooling)	Early and localized increase in MMP-9 concentration within pericontusional brain post-TBI is indicative of BBB damage and edema formation
Petzold et al. (19)	TBI, GCS median 9 (10 TBI, no controls)	3 h–5 d	ECF (contusional and normal appearing brain)	100 kDa MD + standard ELISA and Gel electrophoresis of (sample pooling)	Quantification of specific protein biomarkers (NfH476-986 and NfH476-1026) applicable to *in vivo* monitoring of diffuse axonal injury and neuronal loss in TBI
Lakshmanan et al. (20)	TBI, GCS <8, or a GCS of 9–14 with contusion on CT scan. (2 TBI with normal LPR and 3 with LPR >40.)	<4 d	ECF(contusional and normal appearing brain), serum	20 kDa MD + peptidomics and proteomics approaches based on different MS platforms (MALDI–TOF MS, LC-MS/MS)	Quantification of protein fragments in the ECF. Metabolic distress after TBI is associated with a differential proteome that indicates cellular destruction during the acute phase. This suggests that metabolic stress has immediate cellular consequences after TBI
Jalloh et al. (21)	TBI, GCS <8 (9 TBI)	1–7 d	ECF (normal appearing)	Reverse 100 kDa MD with ^13^C-labeled compounds + *ex vivo* NMR of ^13^C-labeled metabolites	Lower lactate/pyruvate ratio suggests better redox status: cytosolic NADH recycled to NAD^+^ by mitochondrial shuttles. Direct tricarboxylic acid cycle supplementation with 2,3-^13^C_2_ succinate improved TBI brain chemistry, indicated by biomarkers and ^13^C-labeling patterns in metabolites
Orešicˇ et al. (22)	TBI, GCS <8 (5 TBI, no controls)	Acutely after TBI	ECF, serum	100 kDa MD + MS-based metabolomics (GC × GC-TOF-MS)	TBI is associated with a specific metabolic profile in serum which is also reflected in brain ECF (MD samples), which is exacerbated proportionally to the severity of TBI. Top ranking serum metabolites associated with TBI were found highly correlated with their MD levels suggesting possible sensitivity to BBB damage, as well as protective response and altered metabolism post-TBI
**Reference**	**Injury model characteristics**	**Time (after injury)**	**Biosample**	**Techniques employed**	**Key findings**
Dahlin et al. (23)	Model of progressive ICP increase leading to brain death (swine)	<12 h	ECF	100 kDa MD + proteomics by iTRAQ and nanoflow LC-MS/MS (sample pooling)	Definition of *in vivo* performance of a refined MD method, including catheter surface modification, for protein biomarker sampling in a clinically relevant porcine brain injury model. Surface modified MD show improved extraction efficiency for most of the proteins compared to naïve MD catheters

*Patient characteristics describes the injury severity level according to GCS, and the number of patients/controls included in the study. Injury model characteristics describes the experimental TBI model used. Time after injury refers to the sampling time point/s or window. Biosample specifies the sample used for the analysis. Techniques employed describes the technique and/or assay used for the analysis. Key findings highlight any specific insights or notable findings in the papers*.

*BBB, blood–brain barrier; CT, computed tomography; d, day/s; DAI, diffuse axonal injury; DTI, diffusion tensor imaging; ECF, extracellular fluid; ELISA, enzyme-linked immunosorbent assay; GC, gas chromatography; GCS, Glasgow coma scale; h, hour/s; ICP, intracranial pressure; iTRAQ, isobaric tags for relative and absolute quantification; LC, liquid chromatography; LPR, lactate pyruvate ratio; MALDI, matrix-assisted laser desorption/ionization; MD, microdialysis; MMP-9, matrix metallopeptidase 9; MS, mass spectrometry; NAD^+^, nicotinamide adenine dinucleotide (oxidized form); NADH, Nicotinamide adenine dinucleotide (reduced form); NfH, neurofilament heavy chain; NFL, neurofilament light chain; NMR, nuclear magnetic resonance; rhIL-1Ra, recombinant human interleukin-1 receptor antagonist; TBI, traumatic brain injury; TOF, time of flight*.

The clinical use of MD to monitor brain chemistry in neurointensive care has the topic of excellent reviews (24, 25). Clinical bedside MD analyzers using colorimetric enzymatic assays are commercially available for measurement of compounds related to brain energy metabolism, including glucose, lactate, and pyruvate as well as glycerol and the amino acid glutamate, although these are less commonly used clinically. The lactate/pyruvate ratio (LPR) is commonly calculated as an indicator of failing aerobic metabolism and, additionally, has the advantage of being insensitive to recovery fraction since both numerator and denominator are affected approximately equally.

Traumatic brain injury results in well defined metabolic disturbances (i.e., metabolic crisis) characterized by high LPR, high glutamate, and low glucose levels. These metabolic patterns may occur despite adequate resuscitation and controlled intracranial pressure (ICP) (26) and may also be associated with non-convulsive seizures, periodic discharges, and cortical spreading depolarization (27, 28). Metabolic perturbation is related to clinical outcome and is affected by interventions (29–34). Such metabolic traits are of interest since the restoration of metabolic homeostasis is a key goal of intensive care. Elevations in LPR are increasingly clinically accepted as a metabolic marker with a potential to direct therapeutic interventions (25). However, LPR cannot be univocally interpreted as a sign of ischemia. Elevated LPR in the absence of ischemia with normal or high pyruvate concentration has been also described. This may reflect mitochondrial dysfunction (35). LPR elevation with low oxygenation characterizes post-TBI ischemia, whereas metabolic crisis as LPR elevation but with normal or high oxygenation is associated with profound mitochondrial dysfunction but a normal supply of energetic substrate. These two aspects need to be distinguished since augmenting oxygen and substrate delivery in the context of mitochondrial failure will be at best ineffective and could potentially be harmful.

Microdialysis is also currently the only technique for measuring the concentration of putative neuroprotective drugs directly in the brain parenchyma of patients in clinical pharmacological studies (36). Helmy et al. used this technique to demonstrate cerebral penetration of an interleukin-1 receptor antagonist and explore its biological effects by downstream MD sampling of cytokines and chemokines (16). Similarly, Mazzeo et al. used MD to assess the effect of cyclosporin on brain energy metabolism in a randomized, double blind, placebo-controlled study on 50 patients with severe brain injury (37).

Novel, elegant studies have combined ^13^C-labeled MD and detection with *ex vivo* nuclear magnetic resonance (NMR) or *in vivo* MR spectroscopy to provide insights into important biochemical pathways in the human brain (38). Notably, these studies provided evidence that lactate can be used as substrate *in vivo* and that direct tricarboxylic acid cycle supplementation with 2,3-^13^C_2_ succinate improves brain chemistry after TBI (21). Both the pharmacological and labeling studies are examples of novel translational medicine approaches to the mechanistic investigation of putative therapeutic strategies and neuroprotective drugs. Along similar lines, pilot interventional studies have used MD to demonstrate beneficial effects of exogenous lactate supplementation in patients with TBI (30).

Microdialysis may also be used to measure *in vivo* changes in protein biomarker concentrations after TBI (9, 25, 39). Such studies require catheters with a higher permeability cutoff. Colloids such as dextran or albumin solutions are usually added to the standard perfusion fluid to increase both fluid and protein recovery (7, 10). The small volumes collected by standard hourly sampling (at 0.3 µL/min flow rate) are rarely sufficient to measure biomarkers of interest using standard ELISA techniques. Samples may be pooled, but this reduces time resolution (17). Alternatively, high sensitivity ELISA, multiplex approaches (i.e., Luminex technology) and sequential ELISA methods (where, instead of discarding the solution after incubation, a portion is sequentially transferred to further ELISA plates with different antibodies so that unbound analytes can be captured) have been applied in an attempt to overcome this limitation and measure several biomarkers simultaneously with the traditional hourly or two-hourly sampling frequency (7, 8, 18). There is a limit to the number of biomarkers that can be simultaneously measured from the same sample. However, proximity ligation assay (PLA) and proximity extension assay (PEA) technologies are currently being investigated for the simultaneous analysis of more than 100 biomarkers in MD samples (9). PLA technology has superior sensitivity compared to conventional sandwich assays, and it is likely that this methodology will confer a significantly better time resolution.

Recent MD studies have showed that the brain extracellular fluid contains increased concentrations of tau, neurofilament light chain (NF-L), and neurofilament heavy chain (NF-H), all of which are normally cytosolic axonal proteins (8, 17, 19) but which may be released into the extracellular space after TBI as result of axonal injury. Magnoni et al. placed MD catheters in 16 patients with severe TBI and demonstrated the feasibility of quantitative assessment of the axonal proteins NF-L and tau with a 1–2 h time resolution. Importantly, they also found a positive correlation between initial tau levels and poor clinical outcome, likely reflecting the degree of axonal injury (8). Comparable results have also been obtained from measuring tau with standard ELISA, albeit with reduced time sensitivity (17). Similarly, Petzold et al. used high cut-off MD to measure the levels of extracellular fluid NF-H fragments and monitor diffuse axonal injury and neuronal loss *in vivo* after TBI (19). These MD-based assessments of biomarkers of TBI require further validation but could be useful for early phase clinical pharmacodynamic testing of candidate therapeutics targeting axonal injury after TBI. Current technology is not suitable for bedside application. However, improvements to the speed and sensitivity of protein biomarker measurements may change this landscape in the near future. Future studies using methods such as rapid biomarker sampling combined with enhanced analytical techniques and/or novel pharmacological tools could provide additional information on the importance of tau and other proteins in both the acute pathophysiology and long-term consequences of TBI.

Helmy et al. used MD to characterize the cytokine signatures after severe TBI in 12 patients over 5 days using an analytical multiplex approach (i.e., Luminex technology) (7, 40). Forty-two cytokines were measured. Notably, the extracellular fluid concentrations of several of the explored cytokines were significantly higher than plasma concentrations and showed a stereotyped temporal peak, indicating local production.

Several matrix metalloproteinases (MMPs) have also been measured in MD samples from paired MD catheters in pericontusional vs radiologically “normal” sites using a multi-analyte profiling kit. For proteins detection, 8-h samples were pooled together. This study showed that MMP-9 concentrations are increased in pericontusional brain early post-TBI, likely reflecting post- traumatic proteolytic breakdown of the BBB which correlates with hemorrhagic progression and vasogenic edema (18).

At present, clinical MD bedside analyzers employ simple colorimetric assays for the quantification of energetic-related molecules. Bedside MD assessment of protein biomarkers with similarly convenient automated immunoassays would be very attractive for TBI research and translation into clinical practice, when available (41).

### MD Combined with Proteomics and Metabolomics

One new application of MD is in the comparison of protein and metabolic profiles of extracellular fluids in healthy and diseased subjects using proteomic and metabolomic approaches. Compared to classical detection methods, proteomics and metabolomics do not limit the number of molecules simultaneously analyzed. Proteomic studies using MD samples are technically challenging and the MD membrane pore-size represents one intrinsic limitation. Nonetheless, recovery of substances larger than the formal cut-off value has also been observed in proteomic studies of human brain microdialysate using clinical 20 kDa (42) and research 100 kDa catheters in an experimental study (23). In these studies, proteins larger than the formal cut-off value crossed the dialysis membrane to a significant extent although true concentrations in the extracellular fluid are likely to be underestimated. One important technical consideration is that it is impossible to use albumin in the perfusate, which is common using high cut-off MD catheters, as albumin masks the other proteins in the proteomic analysis.

Lakshmanan and coworkers developed another possible application of MD/proteomics by comparing microdialysate samples of TBI patients with normal and abnormal metabolism as evidenced by the LPR threshold of 40 (20). They pursued a diagnostic and biomarkers identification approach by combining peptidomics profiling [by matrix-assisted laser desorption/ionization (MALDI)-MS] with classical bottom-up proteomics profiling (LC–MS/MS). They identified differential proteomic changes (i.e., 13 unique proteins) in the cerebral microdialysate of patients with abnormal metabolism as compared to the control group. These proteins consisted of cytoarchitectural proteins, as well as blood breakdown proteins and a few mitochondrial proteins.

Another interesting application of metabolic MD profiling has been performed by Orešič et al. Applying an untargeted metabolomics strategy based on GCxGC TOF-MS platform, microdialysate was analyzed from 12 samples acquired from 4 TBI patients to study the potential relevance of the serum metabolic profiles in TBI to brain metabolism (22). Interestingly, the top ranking serum metabolites associated with TBI were found highly correlated with their MD levels suggesting possible sensitivity to disruption of the BBB. Among these metabolites were sugar derivatives, metabolites related to energy metabolism as well as several hydroxy-acids. Notably, the medium-chain fatty acids (MCFA, C7–C10) were detected at relatively high concentrations in MD as compared to their corresponding concentrations in blood, while the long-chain fatty acids had lower levels in MD than in blood.

## Multiplex Chemical Profiling

While MD is convenient for measuring temporal profiles, its application is typically limited to sampling relatively small panels of markers that can be recovered from the interstitial space. In reality, biochemical changes following TBI are complex and heterogeneous so a more mechanistically complete description requires the use of techniques capable of the sensitive detection of a great number of analytes simultaneously in any particular biosample of interest. Mass spectrometry (MS) is a key analytical platform capable of multiplex analysis of a wide variety of sample types and underpins the emerging technologies of proteomics and metabolomics. It provides quantitative molecular data and can be used either for the untargeted exploratory identification of hundreds of proteins and metabolites or alternatively in a focused way for the quantification of a small number of known molecular features (either proteins or metabolites) with a sensitivity of down to the parts-per-billion level.

A mass spectrometer measures the mass-to charge ratio [*m/z*] of gas-phase ions. Briefly, the sample of interest is first volatilized and ionized in the ion-source. During the ionization process, molecules may break down into a range of smaller fragments with characteristic charges and masses. The resultant ions are then sorted according to their *m/z* ratio by a mass analyzer. Different ionization techniques and mass analyzers are now available with varying cost and performance (43). There are many different modes of MS analysis ranging from semi-quantitative untargeted methods employing high-resolution instruments or quantitative/targeted analysis (44–46). MS instruments may be coupled to liquid phase pre-separation methods such as liquid chromatography (LC-MS), capillary electrophoresis or ion mobility separation to enhance the dynamic range, sensitivity, specificity, and chemical coverage (47–49). Off-line separation techniques (e.g., CAX-PAGE, SDS-PAGE) can be also employed, especially for protein/peptide separation. Given the chemical diversity of proteins and metabolites and the high sensitivity of this technology, MS has proven its superiority in metabolomics and proteomics. Interested readers are referred to several excellent reviews on the basics of MS and its application in ’*omics* strategies (50).

### Proteomics

Proteomics is the study of the proteome; a set of “all proteins encoded by the genome” (51). Protein expression is highly dynamic, changing in response to environmental stimuli and with the progression of a disease. With advances in technology, neuroproteomics “*a dedicated discipline for the study of the expression, interaction and function of proteins in the nervous system*” has rapidly grown, contributing to the elucidation of the mechanisms of neurological diseases and to the identification of potential biomarkers of injury (52–58). Proteins already identified as having the sensitivity and specificity to be used as clinical biomarkers of TBI include S100B, neuron-specific enolase (NSE), ubiquitin C-terminal hydrolase L1 (UCH-L1), glial fibrillary acid protein (GFAP) myelin basic protein, cleaved tau protein, spectrin breakdown products (SBDPs), and NFL (53, 59–63). Panels of biomarkers may be needed to provide a more accurate and complete description of the pathology (53, 64–66) and could help in patients stratification, selection of treatment strategies, and outcome prediction (53, 56, 67). The application of combined neuroproteomics/neurosystems biology analysis in characterizing dynamic/spatial protein changes and interactions in response to injury is discussed below and cited studies are summarized in Table 2. Excellent reviews are available for complete analysis of these aspects (57, 67, 68).

**Table 2:** MS-based proteomics.

**Reference**	**Patients characteristics**	**Time (after injury)**	**Biosample**	**Techniques employed**	**Proteomics platform**	**Key findings**
Conti et al. (69)	Severe TBI, GCS < 7 (6 TBI vs 6 controls)	<12 h	CSF	Proteomics	2-DE and MALDI-TOF MS	Upregulation of acute phase response proteins (A1AT, HPT1β, α1/2, and tetramer), presence of FDP
Hanrieder et al. (70)	Severe TBI (3 TBI, no controls)	<9 d	CSF	Proteomics	iTRAQ + nanoflow LC coupled off-line to MS/MS	Temporal profile of protein changes in CSF showing changes in acute phase proteins but also brain specific proteins such as GFAP and NSE
Harish et al. (71)	Mild, moderate, and severe TBI (26 TBI patients, 30 TBI autopsy cases)	<4 d (when available)	Brain tissue (biopsy or autopsy)	Proteomics; electron microscopy; energy metabolism, cytokine, antioxidant, and lipid peroxidation assays; western blot	iTRAQ + SCX LC-MS/MS	Contusional and pericontusional tissues exhibit different proteomic signatures
Hergenroeder et al. (64, 65)	Severe TBI, GCS ≤ 8 (11 TBI vs 11 controls)	<3 d	Serum	Proteomics	iTRAQ + LC-MS/MS	CRP and SAA increase in serum after TBI. In contrast RBP4 was reduced
Sjödin et al. (72)	TBI (2 TBI, no controls)	NA	CSF	Proteomics	ProteoMiner protein enrichment technology based on HLL, OFFGEL isoelectric focusing of tryptic peptides, LC-MS/MS	HLL strategy enriched low abundant protein biomarkers in human CSF and increased the number of detected proteins. Well characterized proteins in TBI, i.e., NSE, GFAP, MBP, CK-B, and S-100β were successfully identified
Xu et al. (73)	Severe TBI, GCS ≤ 8 (12 TBI vs 8 controls)	<3 d	Brain tissue (biopsy or autopsy)	Proteomics, western blot	2-plex TMT labeling and LC-MS/MS	Overexpression of proteins involved in glial cell differentiation, immune regulation and apolipoprotein catalysis in the statin pathway
Yang et al. (74)	Severe TBI, GCS ≤ 8 (11 TBI vs 2 controls)	<8 h	Brain tissue (biopsy-frontal cortex-)	Proteomics	2-DE and MALDI-TOF MS	Temporal changes of overall protein expression in TBI (at <3h, 4–6 h and 6–8 h post-TBI) and controls. Significantly changed proteins were mainly involved in metabolism, protein synthesis and turnover, electron transport, cytoskeleton proteins, signaling transduction, stress response, and cell cycle
Gao et al. (75)	Pediatric iTBI (13 TBI vs controls)	<24 h post hospital admission	CSF	Proteomics, western blot	Two-dimensional DIGE, MALDI-MS and LC-MS/MS	HP levels lower in iTBI compared to non-inflicted TBI. PGDS and CC levels was higher in iTBI compared to non-inflicted TBI
Haqqani et al. (76)	Pediatric severe TBI, GCS ≤8 (6 TBI, no controls)	<8 h post injury	Serum	Proteomics, ELISA	ICAT nanoLC-MS/MS	Differentially expressed proteins involved in inflammation, innate immunity, and early stress/defense response (e.g., Toll receptors, signaling kinases, transcription factors, proteases, protein involved in response to oxidative-stress)
**Reference**	**Injury model characteristics**	**Time (after injury)**	**Biosample**	**Techniques employed**	**Proteomics platform**	**Key findings**
Cortes et al. (77)	CCI (rat)	2 d	Brain tissue (pericontusional cortex)	Proteomics	2D-LC/ion mobility IMS/orthogonal TOF-MS	Assessment of protein dynamics and traslocations, including vinculin whose cytosolic traslocation suggests destabilization and retraction of neuronal processes
Crawford et al. (78)	CCI (mouse) mild and severe	24 h, 1, or 3 m	Plasma	Proteomics, ELISA	iTRAQ + LC–MS/MS	Modulation of protein functional clusters related to acute phase response, oxidative stress, and lipid metabolism as function of TBI and in response to TBI*APOE genotype
**Reference**	**Injury model characteristics**	**Time (after injury)**	**Biosample**	**Techniques employed**	**Proteomics platform**	**Key findings**
Kobeissy et al. (57)	CCI (rat)	1–7 d	Brain tissue (ipsilateral cortex)	Proteomics	CAX-PAGE and LC–MS/MS	Decreased abundance of MMIF, aconitase, SOD, NF, and CaM. Increased abundance in complement C3, Pin1, elongation factor 2, and PACSIN
Mehan and Strauss (79),	CCI (rat) in aged, young adults, and juveniles	3 d	Brain tissue (parietal cortex and hippocampus)	Proteomics, western blot, behavioral tests	2-DE and MALDI-TOF-MS	Modulation of 15 proteins isoforms in relation to age and injury in cortex after TBI. Among these: Two isoforms of HSP27, which changed with age, were upregulated in response to injury and showed interactions age*injury; BSA was increased in juveniles only and showed an age*injury interaction; ApoE showed an age*injury interaction
Wu et al. (80)	FP (rat)	4 d	Brain tissue (hippocampus)	Proteomics, western blot	^18^O-water differential labeling and multidimensional tandem LC-MS/MS	Downregulation of 76 proteins at 4 d after TBI mainly related to energy metabolism, oxidative phosphorylation, electron transport chain, calcium signaling and homeostasis. An important downregulation of CANB1 was observed in TBI rats.

*Patient characteristics describe the injury severity level according to GCS and the number of patients/controls included in the study. Injury model characteristics describes the experimental TBI model (and species) used and the injury severity (if available). Time after injury illustrates the sampling time point/s or window. Biosample specifies the sample used for the analysis. Techniques employed describe the technique and/or assay used for the analysis. Key findings highlight any specific insights or notable findings in the papers*.

*2-DE, two-dimensional gel electrophoresis; A1AT, α1 antitrypsin; ADP, adenosine diphosphate; APOE, apolipoprotein E; ATP, adenosine triphosphate; BSA, bovine serum albumin; CaM, calmodulin; CANB1, calcineurin B; CAX-PAGE, cation–anion exchange chromatography-1D SDS gel electrophoresis; CC, cystatin C; CCI, controlled cortical impact; CK-B, creatine kinase B-type; CRP, c-reactive protein; CSF, cerebrospinal fluid; d, day/s; DIGE, difference in gel electrophoresis; ELISA, enzyme-linked immunosorbent assay; FDP, fibrin degradation product; FP, fluid percussion; GFAP, glial fibrillary acid protein; GCS, Glasgow coma scale; h, hour/s; HLL, hexapeptide ligand libraries; HP, haptoglobin; HPT1β and α2/1, haptoglobin 1 β, α2, and α1; HSP27, heat shock protein 27; ICAT, Isotope-Coded Affinity Tag; IMS, ion mobility spectrometry; iTBI, inflicted TBI; iTRAQ, Isobaric Tags for Relative and Absolute Quantitation; LC, liquid chromatography; LC–MS/MS, liquid chromatography-tandem mass spectrometry; m, month/s; MALDI, matrix-assisted laser desorption/ionization; MBP, myelin basic protein; MMIF, macrophage migration inhibitory factor; MS, mass spectrometry; NA, not available; NF, neurofascin; NSE, neuron-specific enolase; PACSIN, protein kinase C and casein kinase substrate in neurons protein 1; PDGS, prostaglandin D2 synthase; Pin1, peptidyl-prolyl cis-trans isomerase A; RBP4, retinol binding protein 4; SAA, serum amyloid A; SCX, strong cation exchange; SDS-PAGE, sodium dodecyl sulphate-polyacrylamide gel electrophoresis; SOD, superoxide dismutase; TBI, traumatic brain injury; TMT, tandem mass tag; TOF, time-of-flight; MS, mass spectrometry*.

Due to the complex and heterogeneous biology of TBI, the analysis and interpretation of the resultant proteome needs to account for both *spatial and temporal changes*. For example, proteins that increase in abundance within the neocortex may decrease within the hippocampus of controlled cortically injured (CCI) rats (79). Moreover, age-related changes in particular proteins are reported after TBI (79), supportive of “*the vulnerability of older patients and resilience of younger ones in recovery after brain injuries*.” Using a label free quantitative platform, Cortes et al. (77) showed that sub-cellular protein translocation and alternative isoform translation occurred in the neocortical tissue from a CCI rat model at 48 h post-injury. They observed the membrane-dissociation of vinculin, a membrane-cytoskeletal protein involved in anchoring actin to the plasma membrane, after injury. Increased cytosolic vinculin might suggest destabilization and retraction of neuronal processes. Furthermore, they highlighted the relevance of protein isoforms (e.g., neurofascin) within the TBI-responsive proteome. Wu et al. (80) used a sophisticated stable isotope ^18^O-water differential labeling and multidimensional LC–MS to profile the proteomic response at 4 days-post TBI in the CA3 sub-region of the rat hippocampus rather than the global hippocampus. They proposed a calcineurin compensatory model conferring protection from extensive TBI-evoked down regulation of energetics and aberrant regulation of proteins responsible of synaptic structures/reorganization, paving the way for the “*identification of novel therapeutic targets for* cognitive rescue in TBI.”

Traumatic brain injury pathobiology evolves over time and *biomarker kinetics* after injury is a critical factor in their clinical interpretation (81, 82). Crawford and colleagues used a quantitative proteomic strategy (iTRAQ-LC/MS) to observe a panel of plasma proteins varying in their temporal profile (24 h, 1, and 3 months) in response to the severity of injury in a CCI mouse model of mild and severe brain injury. At 24 h post-injury, proteomic changes were greater in the mild than severe injury group, suggesting a more complex acute molecular response. In addition, there was greater overlap in protein regulation between the time points in the mild group, indicating that different temporal profile of protein involvement can be associated with the nature of severe injury (78). Bioinformatic analysis of modulated proteins identified particular biological modules associated with TBI, mainly related to the acute phase response, oxidative stress, and lipid metabolism.

Kobeissy et al. (57) recently reported an in-depth description of the dynamic changes in global neuroproteome between acute (1 day post-TBI) and subacute (7 days post-TBI) TBI in rats using the cation–anion exchange chromatography-1D SDS gel electrophoresis (CAX-PAGE) LC–MS/MS platform. Importantly the authors employed systems biology strategies to infer time-dependent changes in cellular pathways caused by TBI. Proteins involved in cell migration, mitochondrial damage, neuronal toxicity, and heat shock response were uniquely altered at 24 h post TBI, while others related to regeneration, axon guidance, axonogenesis, cell growth, and differentiation were solely altered at 7 days post-CCI. Of interest among a total of 19 proteins showing increase in abundance in both acute and subacute TBI samples, the C3 complement component showed a progressive increase over time. C3 is acute phase protein with key systemic and brain local immune regulatory functions; it can be easily measured in biofluids and could represent a promising candidate marker in TBI.

The minimal/lack of availability of brain tissue TBI and control specimens is an obstacle to obtaining direct information on the brain injury processes. Below we describe some of the proteomic studies that have analyzed human brain tissue, CSF or plasma in TBI patients to show how biospecimen selection represents a major challenge in characterizing pathology. Yang et al. (74) investigated how the timing may influence the human brain cortex protein expression profile in 11 patients needing craniotomy. Using a traditional proteomic approach (protein separation by bi-dimensional electrophoresis followed by MALDI-MS-TOF for identification), levels of injury-associated proteins were minimal in the first 3 h after injury, increasing to a maximum at 4–6 h before declining again at 8 h. The functions of the significantly changed proteins were mainly related to metabolism, electron transport, signal transduction, cytoskeletal integrity, stress response, transport, protein synthesis, and turnover. Harish et al. (71) postulated that structural brain characteristics may lead to differences in TBI response and analyzed the proteomic profiles of human contusional and pericontusional tissue (26 individuals) using an advanced technique with improved quantitative accuracy (iTRAQ-LC–MS/MS). The contusional tissue was mainly characterized by altered immune response, and synaptic and mitochondrial dysfunction, while the pericontusional tissue displayed altered regulation of neurogenesis, cytoskeletal architecture, and vesicle proteins. The characteristic signatures of two anatomically adjacent yet distinct regions might help in the mechanistic understanding of injury evaluation. Again, the use of advanced MS-proteomics techniques allowed to identify more than 4,000 proteins in the cerebral parenchyma of post-TBI patients, showing significant alterations in myelin proteins, complement activation, and apolipoproteins (73).

Cerebrospinal fluid and plasma/serum also contain molecular patterns related to the CNS and are alternative biospecimens with higher clinical translational opportunities. CSF is the most representative being in direct contact with the brain particularly in instances where the BBB has been breached. However, protein concentrations vary widely between CSF samples from different TBI patients (55). Plasma/serum samples are easier to acquire than CSF and may have a high overall protein concentration. Furthermore, since blood sampling is safe and well tolerated, normal control data are simple to obtain and such analysis can additionally be applicable to mild TBI where the risks of more invasive techniques are proportionately harder to justify. However, plasma proteins have a wide dynamic range of concentrations, where the presence highly abundant blood-born housekeeping proteins can hinder the discovery of specific proteomic patterns of interest. The analysis of serum proteins is further complicated by extracranial or systemic influences since protein signatures are generally not completely unique to the CNS. Moreover, proteomics patterns found in the CSF are not straightforwardly translatable to blood. Indeed it has been reported that there is only a partial overlap between plasma and CSF proteome (83). One of the first investigations demonstrating the feasibility of a proteomic strategy to study the CSF from TBI patients was performed by Conti et al. (69) using a conventional gel-based proteomic strategy (2-DE and MALDI-TOF-MS) to profile CSF from a small number patients and matched control subjects. Elevated fibrinolysis markers were detected, probably because of blood coagulation after TBI. A similar approach was used to compare the CSF profile from inflicted (*n* = 13) and non-inflicted TBI (*n* = 13) in pediatric patients with severe injury (75). Increased expression of haptoglobin, prostaglandin synthase, and cystatin were found within 24 h of injury. Another study on CSF (72) performed protein enrichment using hexapeptide ligand libraries to reduce protein dynamic range so as to pick-up low abundance CSF proteins. This small proof-of-concept study with only two subjects found proteins associated with degeneration/regeneration, including NSE and GFAP. An interesting time-resolved neuroproteomic study on post-traumatic human ventricular CSF (70) revealed significant temporal changes in the levels of acute phase proteins as well as brain specific proteins (e.g., NSE, GFAP) with a pattern of change between days 3 and 5 indicating sensitivity to secondary events concordant with medical records in a small number of patients. Observations such as these suggest that proteomic analysis may also provide evidence of, and mechanistic insights into, secondary injury. It is noteworthy that the abovementioned CSF proteomic studies also strengthened the use of serum GFAP and NSE as potential prognostic markers for TBI as previously suggested (84, 85).

Although plasma/serum is very clinically convenient, it has been little used in mechanistic-based proteomics studies of TBI. Proteins changes generated in the brain after TBI may potentially migrate into blood but in doing so are greatly diluted. Moreover, plasma and serum proteomics is a challenging task due to the large dynamic range of protein concentration, being 99% of their proteome comprised of 20 highly abundant proteins (86). Despite these hurdles, recent implementation in MS platforms has been essential for the proteomics study of TBI using human plasma/serum.

A feasibility study by Haqqani et al. (76) using isotope-coded affinity tag-based proteomics, identified differences in 95 serum proteins (within 8 h of admission) between six pediatric severe TBI patients and matched controls. These proteins were mainly involved in inflammation and innate immunity indicating a massive defense response. Several of these serum proteins, such as α-spectrin, NSE, tau, and amyloid-A, were likely of brain-origin. In addition, several proteins showed quantitative changes similar to those of S100B, an established serum biomarker of TBI, suggesting that pattern behavior of a group of proteins can help in gaining information about disease characteristics and in the discovery of relevant peripheral TBI biomarkers.

The same technique was used to screen the serum of 11 adult severe TBI patients and matched controls (64, 65). Several proteins altered after injury were in keeping with those identified by Haqqani in pediatric patients, including serum amyloid-A and retinol-binding-protein-4. Interestingly, serum amyloid-A was proposed as indicator of injury severity together with C-reactive protein, whereas retinol-binding-protein-4 was postulated as a predictor of ICP elevation, an important contributor to death/disability following TBI. The authors suggested that possible combination of proteins, each with their own diagnostic window, may help identifying TBI patients who are likely to experience adverse secondary events such as elevation in ICP.

So far, the few clinical proteomics investigations performed on peripheral blood have replicated and strengthened the findings from targeted analysis of specific candidate proteins as TBI biomarkers, recapitulating key features of the neuropathology seen in humans. However, the novel protein patterns identified in these studies are not yet been validated in independent studies.

### Metabolomics

Metabolomics refers to the study of metabolome, which has been defined as “*the complete set of metabolites/low-molecular-weight intermediates (*<*1000Da), which are context dependent, varying according to the physiology, developmental or pathological state of the cell, tissue, organ or organism*” (87). The technical ability to measure thousands of endogenous metabolites simultaneously as signatures that cellular processes leave behind makes metabolomics an emerging strategy in system biology (88, 89). The application of combined metabolomics/neurosystems biology analysis in characterizing dynamic/spatial metabolic signature in response to injury is discussed below and cited studies summarized in Table 3. Hence, metabolomics studies are very promising for understanding complex and multifactorial syndromes and may be a suitable starting point toward personalized medicine.

**Table 3:** MS-based metabolomics.

Reference	Patients characteristics	Time (after injury)	Biosample	Techniques employed	Proteomics platform	Key findings
Dash et al. (90)	Mild (GSC >12) and severe (GSC <8) TBI (mild TBI, *n* = 20; severe TBI, *n* = 20; healthy volunteers *n* = 20)	<24 h	Plasma	MS-based metabolomics, ELISA	LC–MS and GC-MS	Levels of methionine, SAM, betaine, and 2-methylglycine lower in TBI patients compared to controls, indicating decreased metabolism through the transmethylation cycle. Precursors for generation of glutathione, cysteine and glycine also found to be decreased as were intermediate metabolites of the gamma-glutamyl cycle (gamma-glutamyl amino acids and 5-oxoproline). In mild TBI patients, levels of methionine, a-ketobutyrate, 2 hydroxybutyrate and glycine decreased, albeit to lesser degrees than detected in the severe TBI group
Emmerich et al. (91)	Soldiers with mild TBI (*n* = 21), PTSD (*n* = 34), TBI + PTSD (*n* = 13) and healthy controls (*n* = 52)	Chronic time point	Whole blood, plasma	Genotyping APOE, MS-based lipidomics	LC–MS/MS	PL levels decreased in TBI, PSD (moderate-to-severe) and TBI + PSD compared to controls. MUFA-containing PC and PI species decreased in TBI and TBI + PTSD groups but not in PTSD subjects, ether PC levels were lower in PTSD and TBI + PTSD compared to controls. Within PC and PE classes, ratio of AA- to DHA-containing species decreased in mTBI. APOE *ε*4 + subjects exhibited higher PL levels
Jeter et al. (92)	Mild (GCS >12) and severe (GCS ≤8) TBI (mild TBI *n* = 18, severe TBI *n* = 20; healthy volunteers *n* = 20, orthopedic injury without TBI *n* = 15)	<24 h	Plasma	MS-based target metabolomics	LC–MS and GC-MS	Plasma levels of arginine, citrulline, ornithine, and hydroxyproline decreased in severe TBI compared to mild TBI or orthopedic injury. Levels of plasma creatine increased in severe TBI compared to healthy and orthopedic injury subjects. Creatine lower in severe TBI patients that developed high ICP compared to those who did not
Jeter et al. (60)	Mild (GCS <12) and severe (GCS ≤8) TBI	<24 h	Plasma	MS-based metabolomics	LC–MS and GC-MS	Levels of BCAAs (valine, isoleucine, and leucine) decreased in TBI compared to healthy volunteers and patients with orthopedic injury. Only plasma levels of methylglutarylcarnitine were increased after severe TBI. BCAAs plasma levels were similar in mild TBI and orthopedic patients but lower compared to healthy volunteers
Orešicˇ et al. (22)	TBI, GCS ≤8 (5 TBI, no controls)	Acutely after TBI	ECF, serum	100 kDa MD and MS-based metabolomics	GC × GC-TOF-MS	Two medium-chain fatty acids (decanoic and octanoic acids) and sugar derivatives including 2,3-bisphosphoglyceric acid are strongly associated with TBI severity. Serum metabolic profile also reflected in brain ECF (MD samples). Top ranking serum metabolites associated with TBI were found highly correlated with their MD levels suggesting possible sensitivity to BBB damage, as well as protective response and altered metabolism post-TBI
Yi et al. (93)	Moderate to severe TBI (72 TBI patients with cognitive deficits, 31 TBI patients without cognitive deficits, 67 healthy controls)	<12 h	Serum	MS-based metabolomics	GC-MS	A serum metabolites panel consisting of serine, pyroglutamic acid, phenylalanine, galactose, palmitic acid, arachidonic acid, linoleic acid, citric acid, and 2,3,4-trihydroxybutyrate was identified to be able to discriminate between TBI patients with cognitive impairment, TBI patients without cognitive impairment and healthy controls
**Reference**	**Injury model characteristics**	**Time (after injury)**	**Biosample**	**Techniques employed**	**Proteomics platform**	**Key findings**
Abdullah et al. (94)	Severe CCI (mouse)	3 m	Hippocampus, cortex, cerebellum (left and right) and plasma	Behavioral tests, MS-based lipidomics	LC-MS/MS	Total PC-, SM-, and PE-species increased in hippocampus but decreased in cortex and cerebella of TBI mice compared to controls. Total PL levels decreased in plasma of TBI mice. Ether-PC in the cerebella and ether-PE in cortex decreased in TBI mice. PUFA-containing PC and PE species, particularly ratios of DHA to arachidonic acid decreased in the hippocampi, cortex, and plasma of TBI mice
Bahado-Singh et al. (95)	WDI (mouse)	4 h and 1 d	Serum	Target quantitative metabolomics	Biocrates platform with FIA and LC–MS/MS	Thirty-six of 150 measured metabolites were different in TBI compared to control mice. Temporal changes (from 4 to 24 h) were observed in 56 metabolites after TBI. The combination of six metabolites achieved complete accuracy for distinguishing early TBI (4 h) from late TBI (24 h) with spermidine as the most discriminating biomarker. Affected pathway included arginine, proline, glutathione, cysteine, and sphingolipid metabolism pathways
Emmerich et al. (96)	CHI (mouse)	1 d; 3, 6 m; 1 and 2 y	Plasma	MS-based lipidomics, ELISA	HILIC LC-MS/MS	PC, PE, PI, and SM levels decreased with aging. PC, LPC, PE, LPE and PI (but not SM) were decreased at 3 months post-TBI, and all classes were decreased at 24 months post-TBI compared to controls. Total lipid peroxidation was elevated at 3 months post-TBI compared to control when PUFA levels were decreased.
Sheth et al. (97)	mild and severe TBI (rat), tMCAo (mouse), acute stroke (9 stroke patients, 5 stroke-mimic patients)	TBI and tMCAo: 4 h, 1, 2, and 7 d. Stroke patients: within 3 h from the first symptom	brain tissue (only mice and rats), plasma, sphingolipids extraction	TTC, immunostaining, MRI, target MS-based lipidomics	LC-MS/MS	*TBI*: 56 SLs species were present at higher concentration in brain compared to plasma. SL increased in plasma from 6 to 24 h post TBI with SM increase proportional to CCI severity. *tMCAo*: 45 SLs species were present at higher concentration in brain compared to plasma. SL increased in plasma from 6 to 24 h post tMCAo with SM and Cer showing the largest relative changes. SM + Cer defined the SL score. *Stroke patients*: within 3 h post-stroke, SL score was higher than in stroke-mimic patients. The SL score correlated with the volume of ischemic brain tissue by MRI

*Patient characteristics describe the injury severity level according to GCS, and the number of patients/controls included in the study. Injury model characteristics describe the experimental TBI model used and the injury severity (if available). Time after injury illustrates the sampling time point/s or window. Biosample specifies the sample used for the analysis. Techniques employed describe the technique and/or assay used for the analysis. Key findings highlight any specific insights or notable findings in the papers*.

*AA, arachidonic acid; APOE, apolipoprotein E; BBB, blood–brain barrier; BCAAs, branched-chain amino acids; CCI, controlled cortical impact; Cer, Ceramides; CHI, closed head injury; d, day/s; DHA, docosahexaenoic acid; ECF, extracellular fluid; ELISA, enzyme-linked immunosorbent assay; FIA, flow injection analysis; GC, gas chromatography; GCS, Glasgow coma scale; h, hour/s; HILIC, hydrophilic interaction chromatography; ICP, intracranial pressure; LC, liquid chromatography; m, month/s; MD, microdialysis; MRI, magnetic resonance imaging; MS, mass spectrometry; MUFA, mono-unsaturated fatty acids; PC, phosphatidylcholine; PE, phosphatidylethanolamine; PI, phosphatidylinositol; PL, phospholipids; PTSD, post-traumatic stress disorder; PUFA, polyunsaturated fatty acid; SAM, S-adenosylmethionine; SLs, sphingolipids; SM, sphingomyelin; TBI, traumatic brain injury; tMCAo, transient middle cerebral artery occlusion; TTC, 2,3,5-triphenyltetrazolium chloride; WDI, weight drop injury; y, year/s*.

There are two general analytical approaches to metabolomic analysis. *Targeted* metabolomics refers to the detection and precise quantification (nM, or mg/mL) of a small set of known compounds. It is a hypothesis-driven approach, where then set of metabolites related to one or more pathways is already defined. A limitation of the targeted approach is that it requires the compounds of interest to be known *a priori* and to be available in their purified form. The *untargeted* approach (“metabolite fingerprinting”) is instead data driven and is used for complete metabolome comparison (i.e., as many metabolites as possible are measured) (88).

The most common analytical instrumentations used in metabolomics are MS or NMR spectroscopy. Both techniques have advantages and disadvantages, briefly mentioned here and the reader is referred to a number of excellent review articles describing the technology involved (88, 98, 99).

Mass spectrometry is sensitive to ionizable compounds down to the picomolar range. Such high sensitivity makes MS-based methods powerful tools especially in targeted studies when the absolute quantification of groups of metabolites is the goal. In exploratory approaches, it can be challenging to identify hundreds of metabolites simultaneously with the same efficiency for technical and computational reasons (100). NMR spectroscopy is used to identify and quantify compounds in solution that are MR detectable. Despite being less sensitive than MS (micromolar range), NMR is sample-non-destructive and it is highly reproducible (101).

The study of small molecules that enter biochemical pathways has a long history in TBI research. For example, glucose and lactate dynamics have been studied with respect to TBI outcome, disease progression, and mechanisms (102, 103). What distinguishes contemporary studies is the technologies available for the simultaneous analysis of many metabolites in a given sample. Metabolomics has been applied to the diagnosis/prognosis of many diseases from cancer to diabetes to cardiovascular pathologies (104–106) and is now an active area of research in TBI. In this section, we will focus on most recent TBI metabolomics investigations based on MS technologies applied to preclinical models and clinical settings as well.

Although, in recent years, there has been a much needed influx of TBI metabolomics studies due to various confounding variables (e.g., selection biases, genetics, diet) and practical limitations (e.g., chronic effects of repeated mild TBI and ethical considerations), animal models are still important for the exploration of metabolic signatures of TBI for the development of potentially translatable approaches in both the detection and clinical monitoring of patients.

Since lipids and phospholipids (PLs) play important roles in the structural and functional integrity of neuronal membranes, vesicular trafficking and in neuroinflammatory responses (91, 107–109), many investigators have focused in identifying lipid abnormalities associated with TBI (“lipidomics”).

Disturbances of lipid profiles in a fluid percussion injury (FPI) rat model of TBI have been known since the 1990s (110) and are a consequence of activating the phospatidylinositol 4,5-bisphospate (PIP2) signal transduction pathway in injured brain. Similar findings were reported for another rat CCI model where free fatty acids (FFA) and diacylglycerol (DAG) levels were increased in the sensorimotors cortex and cerebellum of injured rats compared to sham animals (111).

Using a similar model of TBI, Abdullah et al. (94), used LC–MS to investigate the PLs profiles in mice 3 months post-injury and reported decreased ether phosphatidylethanolamines (ePE) levels in the cortices and plasma of injured animals. Moreover polyunsaturated fatty-acid (PUFA)-containing phosphatidylcholine (PC) and PE species were lower in the hippocampus, cortex, and plasma of injured mice, thus indicating that TBI affects both brain and plasma PL levels, with plausible roles in the inflammatory response.

Recent advances in MS techniques have allowed the development of approaches focused on specific lipid subsets permitting in-depth analysis of lipids species (112). By targeting lipid profiling to sphingolipids (SL), Sheth et al. (97) showed large increases in many circulating SL following TBI in rats, with sphingomyelins (SM) being the most prominent species and larger lesions produced proportionately larger increases. Since increases in many SLs species were also noted in plasma of mice after stroke and a linear correlation between circulating SLs and infarct volume was observed in stroke patients with neurological deficits, the authors suggested that lipid subset can be used as biomarkers of brain injury.

Phospholipid abnormalities may persist long after the initial injury, as reported in a closed head injury (CHI) mouse model of TBI (96). By using HILIC LC/MS techniques, it was shown that saturated, mono-unsaturated fatty acids (MUFA) and PUFA were differently regulated over time and ePE species were elevated at 24 h after TBI and decreased relative to controls at chronic stages (3, 6, 12, and 24 months post-injury). Such longitudinal profile could potentially serve not only as surrogate diagnostic marker but also might enable the discovery of molecular targets for precision medicine.

Tracking changes in the serum metabolome to profile on-going damage was also addressed recently by Bahado-Singh et al. (95) using a targeted quantitative MS platform in a mouse model of TBI. Arginine and proline pathway, glutathione metabolism, SL metabolism, and polyamine pathways changed significantly over time suggesting their potential utility for ascertaining the timing of TBI and for monitoring the degree of oxidative injury and associated neuronal damage.

Clinical evidence of PL involvement in TBI has been available for some time (113, 114), in that increased glycerol (an indicator of PL degradation) concentration is found in the CSF dialysate from brain-injured patients and during the first 24 h in subjects with an unfavorable outcome after severe TBI (115). A cross-sectional study reported that patients with TBI had increased PL within their CSF relative to control subjects (116). Moreover, CSF levels of PC and PE were enhanced in patients who did not survived following severe TBI compared to those who survived (115). Although not really lipidomic studies, they pointed to a possible association between brain PL disturbances and TBI and paved the way to more sophisticated analyses to deep insight the role of lipid classes in TBI pathophysiology.

Detectable PL disturbances in the CSF suggests that such signals might also be detected in peripheral biofluids. In reality, the interplay between brain and systemic lipid metabolism is complex. In fact, the brain can synthesize saturated (SFA) and MUFA containing PL species; whereas PUFA are transported from blood to brain, where they serve many functions (e.g., membrane repair and lipid mediators) (117).

By using an MS-based lipidomic platform able to qualitatively and qualitatively analyze several 100 PL species and their degree of saturation, Emmerich et al. (91) screened a large cohort of military personnel (120 subjects) and found that PL species profiling, together with APO-E genotyping, was helpful to differentiate mild TBI and post-traumatic disorder, whose diagnosis is often difficult because of overlapping symptomatology.

Interestingly, exploiting a sophisticated metabolomic platform based on 2-dimensional gas chromatography coupled to high resolution MS (GCxGC-TOF-MS) Orešič et al. (22) showed that two MCFAs increased in serum from a large set of TBI patients (144 subjects) and this was strongly associated with the severity of TBI. The accumulation of MCFA might be particularly intriguing due to their role in fatty acid oxidation disorders (118). Thus, MCFA variations may inform on mitochondrial dysfunction in TBI. Sugar derivatives and hydroxyl acids were also upregulated in the serum of TBI patients, suggestive of BBB disruption and disturbed energetics. Outcome prediction was further improved when the metabolite dataset was combined with clinical and imaging variables.

As expected, changes in several other circulating metabolites may also contribute to the “TBI metabotype.” Differences in plasma levels of l-arginine and its key metabolic products have been found between severe and mild TBI patients (92). l-arginine is involved in metabolic pathways critical for cerebral blood flow (CBF) regulation and extracellular matrix (ECM) remodeling (119), thus authors speculated that alteration in circulating ariginine found in severe TBI patients may contribute to brain injury such as decreased CBF and collagen synthesis.

Cognitive impairment is one of the most significant TBI-associated disabilities (120). TBI patients with and without cognitive impairment, and healthy controls could be discriminated by a panel of serum metabolites (serine, pyroglutamic acid, phenylalanine, galactose, palmitic acid, arachidonic acid, linoleic acid, citric acid, and 2,3,4-trihydroxybutyrate), identified by GC-MS, although the study was small (93). Nevertheless, post-TBI cognitive impairment was associated with altered metabolism of amino acids, carbohydrates, and lipids. The detrimental role of lipid dyregulation during neurodevelopment and repair after TBI was suggested by the altered levels of arachidonic acid (increased) and palmitic acid (decreased) TBI patients with cognitive impairment (121, 122). Indeed, arachidonic acid, a pro-inflammatory and oxidative lipid species, was also found to be significantly increased in serum from TBI subjects (123, 124) and was associated with platelet dysfunction following TBI (125).

A targeted metabolomic study showed that branched chain amino acids (BCAA) concentration was decreased in TBI patients (60) although this was not confirmed by Orešič et al. (22) probably due to the high variability in BCAA levels. A further targeted metabolomic investigation, based on a combination of LC–MS and GC-MS techniques, reported that severe TBI patients have low levels of plasma methionine and its metabolic products (e.g., S-adenosylmethionine, 2 methylglycine) and their decrease may contribute to brain injury pathology (90). However, caution has to be exercised when considering circulating dietary amino acids such as methionine, when diet intake is not controlled for.

## Novel Spatially Resolved Technologies

The proteomic and metabolomic/lipidomic techniques described thus far rely on global measurements from a particular body compartment. However, chemical changes occur over spatial length scales that can reach down to the sub-cellular level and are dynamic over time. To further understand biochemical events at a local tissue or even cellular level, a spatial mapping is required and imaging plays an important role. Traditionally, this has been achieved via immunohistochemical or other labeling techniques. However, in the context of the ’*omics* paradigm presented, such labeling techniques are restricting as they typically probe only a few different species at a time and can only be applied to *ex vivo* tissue sections. A label-free spatially resolved technique yielding pleiotropic chemical information would be highly desirable, particularly if this could be achieved in a non-destructive way that could ultimately be deployed *in vivo*.

### Mass Spectrometry Imaging (MSI)

For accurately understanding the pathological condition of a tissue such as brain, which has many functional compartments it is not only necessary to determine the spectrum of molecular features involved in the processes, but also to visualize their spatial distribution within the tissue. MSI is one of the latest rapidly growing techniques for the identification and spatial localization of molecules in tissues (126, 127). MALDI is the most common ionization techniques used in MSI. Ions are produced directly from a tissue slice coated with MALDI matrix and sequential masses are acquired across the tissue surface (128). In a typical MALDI-MSI experiment “*a tissue section is deposited on a steel plate, sprayed with a matrix solution and analysed by MALDI-MSI. The distribution of biomolecules on a tissue section can be readily visualized in two-dimensions, assigning to each pixel the ion intensity specific for the molecule under study*” (129).

Mass spectrometry imaging permits the simultaneous visualization of many types of molecules such as small molecule drugs, metabolites, peptides, proteins, and lipids. Since MSI is a label-free technique that provides the possibility to combine tissue histological data with MS ones it represents a powerful tool for visualizing simultaneously the distribution of molecules in relation to tissue heterogeneity and its pathological status (130).

Experimental TBI studies revealed that the cortex, hippocampus, and thalamus are selectively vulnerable to injury (131–134). Previous studies reported alterations in the lipid profile as an important contributor to this vulnerability and the evolution of secondary damage in TBI (135–137). Consequently, efforts have been made in determining spatial distribution of altered lipid profile in response to brain injury using MSI.

In a study assessing lipid changes in response to blast-induced mild TBI, major increases in ganglioside GM2 were noted in the hippocampus, thalamus, and hypothalamus after a single blast exposure (138). A concomitant decrease in ceramides was also noted in the same study. Hankin et al. (139) showed changes in lysophosphatidylcholine, PC, PE, and SM that were specific to their adduct ions in response to ischemia/reperfusion injury in the brain. By using an innovative silver nanoparticle protocol, Roux et al. (140) imaged rat brain lipids over time (1, 3, and 7 days), in a CCI model of TBI. Their results indicated that increases in SM and ceramide were already detectable 1 day post-TBI in the injured cortex. In addition, changes in derivatives of DAGs, cholesteryl esters, PE, and PI were noted at later time points (days 3 and 7 post-TBI). The kinetic differences in lipid classes might give an insight into the time-course of injury response and remodeling of injured brain tissue.

A biochemical map of critical mitochondrial PL species of cardiolipin (CL) in several anatomical brain regions from a CCI rat model of TBI was recently provided by Sparvero et al. (141). The authors demonstrated that regional/spatial CL decrease occurred not only within the contusion but also in the hippocampal and thalamic regions, distant from the site of injury. They proposed that the specific decreases in CL, which will ultimately influence mitochondrial efficiency, “*may constitute an upstream mechanism for CL-driven signalling in different brain regions as an early response mechanism and may underlie the bioenergetic changes after TBI*.”

### Optical Spectroscopy

Optical spectroscopy reveals a chemical fingerprint of any given sample, based on specific absorption of different colors of light by different molecular bonds. Analogous to masses held together by springs, energy absorbed from incident light by molecular bonds can generate vibrations and rotations, such as stretching, torsion, or bending, depending on the molecule. The frequencies of such “modes” of oscillation are determined by the nature of the inter-atomic forces and are, therefore, characteristic to the bond. The frequencies and geometries of permissible modes are governed by quantum mechanical considerations and are dependent on the detailed electronic configurations and symmetry of the bonds in question. Cellular components such as lipids, proteins, and nucleic acids consist of many bonds and, thus, as a whole, provide a rich chemical fingerprint, particularly for frequencies in the infra-red (IR) part of the electromagnetic spectrum. Optical spectroscopic techniques are advantageous for chemical analysis as they are non-destructive; can be easily integrated with imaging; and, in principle, can be easily translated to *in vivo* applications.

Raman spectroscopy (RS) provides a chemical fingerprint based solely on vibrational interactions with tissue. The chemical information obtained from RS is similar to that obtained by IR spectroscopy (although symmetry and charge considerations mean that not all Raman active modes are IR active and *vice versa*). The Raman effect is very weak but high sensitivity can be achieved locally by focusing the probe beam via a microscope arrangement onto the sample being analyzed and using high sensitivity detection instrumentation. The relatively low-cost availability of stable and compact high intensity laser light sources has greatly facilitated this technique. However, heating of the sample limits the signal to noise improvements that can be achieved by simply increasing the incident laser power for biological specimens.

Raman spectroscopy has been applied to models of brain injury from radiation (142) and penetrating trauma (143) as well as in a model of peripheral nerve injury (144). More recently, RS has been used to characterize chemical progression and resolution in a model of focal TBI (145). Raman spectra were recorded from various regions in the cortex in contusional and pericontusional tissue as well as from the contralateral hemisphere in specimens taken at 2 and 7 days post injury as well as from sham controls. Significant differences in chemical spectra were seen. Most significantly, there were differences in protein signal, including strong changes from heme associated with acute hemorrhage at the contusion site at 2 days. Hemorrhagic conversion is an important and devastating process, and one that may directly contribute to cytotoxic and oxidative damage (146). This heme signal was found to have resolved by 7 days consistent with the phagocytic clearance of hemoglobin and heme by macrophages/microglia. Furthermore, the authors found differences in lipid composition—in particular an elevation in cholesterol signal relative to PL at both 2 and 7 days at the site of the contusion and in pericontusional tissue. This lipid sensitivity of RS is particularly attractive given the importance of lipid metabolism in neuronal degeneration and repair and, in particular, the upregulation of cholesterol transport after injury since this material is critical for cellular repair. The ratio of particular Raman peaks relating to the relative concentration of β-sheet protein has previously been suggested as being sensitive to amyloid and has been studied in “cleaner” systems such as the eye lens (147). The authors observed changes in this signal after TBI also. However, there is generally substantial overlap between lipid and protein signals, which can make these observations difficult to interpret with certainty. Acquisition of spectra from pure reference compounds can reduce uncertainty and assist with identification of some specific lipid and protein contributions, such as those described above. Furthermore, overall changes in lipid and protein concentration are easily detectable and relevant to the disease biology. This lipid and protein sensitivity makes RS a promising technology for research into key pathways of interest after TBI. A key advantage of RS is the potential for imaging and it is possible to demonstrate spatial changes in both protein and lipid chemistry.

While RS is a multiplexed technique, the related technique of coherent anti-stokes Raman scattering (CARS) allows the targeted examination and imaging of particular Raman bands with high sensitivity and has been employed in areas allied to TBI, such as the study of myelin degradation processes where it has revealed a calcium-dependent pathway in a spinal cord model of demyelination (148).

Raman spectroscopy is a label-free and non-destructive technique. RS and allied techniques such as CARS show considerable promise as both untargeted and targeted chemically sensitive imaging techniques. If the spatial resolution is sacrificed, in principle, it is possible to miniaturize the equipment and perform RS *in vivo* via implanted fiber-optic probes offering the possibility of a continuous multiplex assessment of chemistry. Although the RS signal is weak, it increases with the fourth-power of incident light frequency and so signals using green or blue laser illumination are far stronger and have better signal to noise than can be achieved with conventional red or IR illumination. However, these frequencies can also excite tissue autofluorescence, which interferes with the baseline of the Raman spectrum making quantitative analysis more difficult. Although a nuisance for RS, flavoproteins such as NADH exhibit strong redox-sensitive fluorescence signals offering the possibility of simultaneous interrogation of the status of the electron transport chain.

## Future Challenges in TBI ’Omics: Opportunities for System Biology

The flurry of research in TBI ’*omics* over the last decade is promising but many issues have to be addressed before deciphering complex molecular patterns that define TBI biochemistry. The limited sample sizes studied to date, differences in sample collection protocols, in outcome measures and limited number of longitudinal clinical designs are hindering the assessment of patient ’*omics* trajectories. A better tracking of protein dynamics and range of post-translational signaling events are also needed. The development of multiplexing quantitative targeted proteomics would help in choosing the most specific candidates from the wide range of potential protein biomarkers found in the literature and in moving into clinical validation and assay development. Such approaches could complement the use of conventional proteins biomarkers assays and provide a new framework to develop and optimize interventions. The analytical technology will need to be further refined if clinical applications are to ultimately be realized as current experimental techniques are too cumbersome for bedside use. Cost is a further consideration: experimental assays are expensive both in terms of equipment and consumables. While this is perhaps less of a limitation in the intensive care unit where treatment costs are already significant, it is a particular problem for applications in mild/moderate TBI and the benefits in terms of treatment would need to be significant in order to be justifiable with current technology. Having said this, bioassay technology is advancing continuously and there are many potential applications of clinical metabolomics and proteomics outside TBI driving advances, so the future landscape is likely to look very different.

While metabolic changes are fundamental to TBI pathophysiology and metabolomic signatures may contain prognostic information, it remains unclear if these metabolic changes are causative or just a consequence of TBI pathophysiology. Moreover, further studies are needed to determine to what extent observed metabolic profiles are brain specific or whether they reflect interactions with systemic changes.

The fast-growing ’*omics* domain have been facilitated by advances in analytical technology such as various MS modalities or future optical spectroscopic methods and are generating a massive amount of molecular data, offering the identification and quantification of hundreds of molecules involved in diverse cellular pathways and, therefore, their consequent phenotypes. The complexity of biological systems and large number of variables coupled with the relatively low number of observations (e.g., samples) make integrative analyses challenging. One of the greatest challenges is that of TBI bioinformatics. The development of easy-to-use informatics software was required to leverage genomic data and similar information technology developments will need to take place before proteomic and metabolomic/lipidomic data can be fully exploited. Progress is being made in developing novel analytical techniques (149, 150). However, the challenges are significant as the data are far more complex than is the case for genetics. A paucity of specialized mathematical, statistical, and bioinformatics tools and the need of considerable computational effort hampers rapid progress in the field (151–153).

Recently, Feala and colleagues elegantly addressed this issue in TBI research (53) with the suggestion that existing TBI data sets available from the literature could be exploited. They compiled a list of proteins candidates from the TBI literature and by applying network and pathway analysis, were able to generate candidate new biomarkers. From their integrative network analysis, the protein kinase ABL1 was particularly appealing being already known as tractable target by imatinib in chronic myeloid leukemia.

Functional interpretation of proteins or metabolites usually includes enrichment analysis and pathway analysis that often overlap as both work by comparing significant features, identified by different statistical workflows, to predefined knowledge databases. To this purpose, many tools exist either free (e.g., MetaboAnalyst,) or on the market (e.g., MetaCore™ from Thompson Reuters; IPA from Qiagen) with their own advantages and drawbacks.

One possibility for a truly comprehensive characterization of TBI might consists in simultaneously monitoring the levels of transcripts/proteins/metabolites from the same study population and in combining the resulting multilevel data to infer the dynamics of the underlying biological networks.

Multiomic integration—the full knowledge of the proteome/metabolome and its spatio-temporal evolution after TBI—would be a powerful insight into the underlying pathobiology. However, the goal of omics techniques is not simply to catalog all the transcripts, proteins, and metabolites; rather it is to understand biological mechanisms. To do this, it is necessary to map metabolomic and proteomic data onto known pathways.

The abovementioned software (MetaCore; IPA) and others, such as InCRoMAP (154), 3Omics (155), IMPaAa (156), already allow the mapping of different molecular features (gene, miRNA, protein, metabolite) into biological pathways as networks of interconnected nodes, allowing their visualization for the easy identification of relationships and hidden patterns for hypothesis generation. Recently, network approaches have been used to relate genomic and proteomic signatures in cellular pathways pertinent to experimental TBI-related cellular systems (157).

However, there are still open challenges in ’*omics* data integration (including dimensionality, heterogeneity, data source and processing, computational power, and capacity) that need to be faced to take effective advantage of new experimental multilayer data. Moreover, given the complexity of TBI, only a comprehensive and integrated analysis of molecular data with clinical measurements may help to plan an early and appropriate intervention in TBI. In this context, incorporation of ’*omics* data into clinical practice may, thus, provides a means to establish new therapeutic targets and to follow individual patient response to therapy. Data mining and machine learning approaches are now representing a very powerful tool since they can be used to develop classification models and to elucidate early multilevel markers signatures which could reveal the biological pathways involved in disease progression (101, 158–160).

Future sophisticated systems biology approaches may allow TBI researchers to make biological sense of the vast quantity of data now available in order to identify novel therapeutic targets and assess the effectiveness of treatments or alternatively, through correlation with population cohorts, to realize a precision medicine approach through the intelligent targeting of interventions to patients in whom they may be most effective.

## Author Contributions

AE and EZ conceived and coordinated the review group. SM and GV drafted section 1, RP drafted section 2, and SB and JS drafted section 3. All authors read and approved the final manuscript.

## Conflict of Interest Statement

The authors declare that the research was conducted in the absence of any commercial or financial relationships that could be construed as a potential conflict of interest.
